# Radiologic Modalities and Response Assessment Schemes for Clinical and Preclinical Oncology Imaging

**DOI:** 10.3389/fonc.2019.00471

**Published:** 2019-06-04

**Authors:** Farshid Faraji, Ron C. Gaba

**Affiliations:** ^1^University of Illinois College of Medicine, Chicago, IL, United States; ^2^Department of Radiology, University of Illinois Health, Chicago, IL, United States

**Keywords:** Radiology, imaging, trial, standards, large animal model

## Abstract

Clinical drug trials for oncology have resulted in universal protocols for medical imaging in order to standardize protocols for image procurement, radiologic interpretation, and therapeutic response assessment. In recent years, there has been increasing interest in using large animal models to study oncologic disease, though few standards currently exist for imaging of large animal models. This article briefly reviews medical imaging modalities, the current state-of-the-art in radiologic diagnostic criteria and response assessment schemes for evaluating therapeutic response and disease progression, and translation of radiologic imaging protocols and standards to large animal models of malignant disease.

## Introduction

Clinical trials are an essential component of the drug development process, and are used to aid in the systematic assessment of newly developed agents. This regulatory framework was developed in order to create a standardized methodology for evaluating drug safety and efficacy. Given that a clinical trial for the development of a new drug requires an investment of 10 years and well over $1 billion—with greater cost to drug developers and public health risk to patients should a drug go to market and subsequently be deemed unsafe or ineffective—drug developers are eager to optimize trials in order to improve early identification of drug failure and minimize confounding variables, biases, and statistical errors (1). Outcome measures used to inform decision-making vary depending on trial phase, whereby early phases evaluate safety and potential efficacy of a drug, and later phase trials assess effect on clinical outcomes compared to a placebo or standard of care. Regardless of trial phase, radiologic imaging has emerged as a valuable tool for assessment of drug efficacy in oncology clinical trials due to the ability to longitudinally assess tumor size and viability in a non-invasive and standardized manner. The two-fold purpose of this review article is to: (1) provide an overview of medical imaging and tumor response assessment standards for clinical oncology trials in order to provide necessary context on imaging needs for preclinical cancer trials and to offer example systems from which to develop animal based imaging standards and schemes in the future; and (2) to describe the applicability and translation of radiologic imaging protocols and standards to preclinical large animal models of malignant disease, with a focus on unmet needs.

## Imaging Modality Overview

A synopsis of radiologic imaging modalities relevant to oncology clinical trials—spanning computed tomography (CT), magnetic resonance (MR) imaging, ultrasound, and positron emission tomography (PET)—is presented in Table 1.

**Table 1:** Overview of radiologic imaging modalities relevant to oncology clinical trials.

**Parameter**	**CT**	**MR imaging**	**PET**
Imaging basis	Ionizing x-radiation	Non-ionizing radiofrequency radiation	Ionizing radiation (positron emitting radiotracer ± x-radiation for concurrent low dose CT scan
Preparation	None needed	None needed	Fasting × 6 h (diabetic patients require close glucose monitoring); no strenuous activity before study to avoid muscular deposition of radiotracer;
Contraindications	Avoid in pregnant women and pursue with caution in women of childbearing age	Avoid in patients with metallic implants (e.g., pacemakers, aneurysm clips)	If concurrent low dose CT scan obtained, avoid in pregnant women and pursue with caution in women of childbearing age
Acquisition time	Short (5 min)	Long (30–90 min)	Long (60 min for radiotracer to reach target tissue + 30 minutes for imaging)
Contrast material	Iodinated contrast material; may cause contrast-induced nephropathy	Gadolinium contrast material; may cause nephrogenic systemic fibrosis	None

*CT, computed tomography; MR, magnetic resonance; PET, positron emission tomography*.

### Computed Tomography

**Figure 1:**
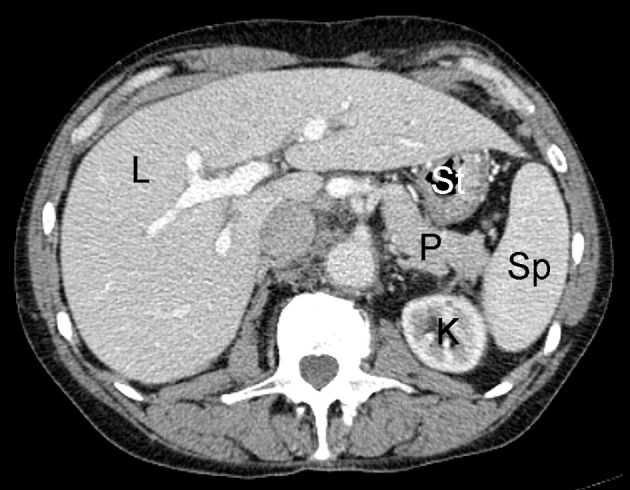
Example of normal contrast-enhanced abdominal CT scan in human patient; L, liver; K, kidney; P, pancreas; St, stomach; Sp, spleen.

Introduced in 1972, CT was the first non-invasive radiologic imaging technique allowing for tomographic imaging without superimposition of neighboring anatomic structures onto one another. This imaging technology operates through the acquisition of x-ray images spanning different angles across a single axis of rotation, and uses computer algorithms to reconstruct these planar projection images into cross-sectional slices (Figure 1). The x-ray imaging technology which this imaging modality is based upon measures the attenuation of high energy photon beams transmitted through a subject. Measurement of the attenuation coefficient allows for the differentiation of tissues based on their density, as tissues with high density (such as bone) will attenuate a higher proportion of a photon beam than those with lower densities (such as muscle or fat). Detecting these subtle differences in tissue density is helpful in the detection of tumors, as disordered neoplastic growth may result in changes in tissue density (2). Modern CT imaging has many advantages, including the ability to image large volumes with sub-millimeter resolutions in a short time span, and the capability for multi-planar reformatting of images in sagittal and coronal views after imaging data has been acquired. CT imaging does carry small risk, however, as the exposure to high doses of ionizing radiation may increase the probability of developing some cancers (3–5). However, advances in CT instrumentation, detector technologies, and image reconstruction algorithms have allowed for the acquisition of high quality images with significant radiation dose reduction (6–8).

### MR Imaging

**Figure 2:**
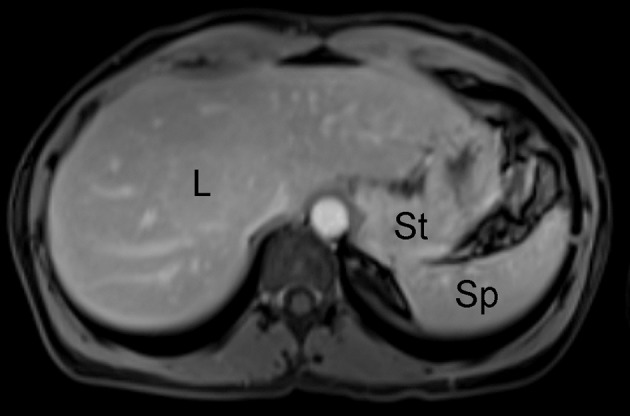
Example of unremarkable contrast-enhanced abdominal MR imaging study of the abdomen in human patient; L, liver; St, stomach; Sp, spleen.

MR imaging is a widely available imaging technique that uses a magnetic field and radiofrequency pulses to non-invasively generate cross-sectional images using the inherent magnetic properties of the human body. Initially applied to human imaging in 1977, this modality utilizes various pulse sequences, which are time varying gradient magnetic fields coordinated with radiofrequency pulses. These pulse sequences take advantage of tissue specific properties of magnetic relaxivity (termed T1 and T2) in order to generate image signal and contrast (Figure 2). These pulse sequences can be implemented in a variety of ways, and can be used to selectively null signal from fat and to measure properties such as the diffusion of water within a tissue, among numerous other applications. Unlike CT, MR imaging employs radiation in the radiofrequency range which is non-ionizing, making it more suitable for repeat imaging sessions. MR imaging does have limitations, however, as it carries risk for patients with metallic implants such as pacemakers, synthetic valves, orthopedic prostheses, and aneurysm clips due to the possibility of dislodging these implants from interaction with and motion because of the magnetic field (9). There is also the risk of heating of tissues adjacent to implants due to deposition of radiofrequency energy (10). Another disadvantage of MR imaging is the lengthy imaging time required to conduct high resolution MR imaging protocols, which can increase the possibility of patient motion and lead to image quality degradation. However, the adoption of image acceleration techniques (such as parallel imaging and compressed sensing) have allowed for reduction of these long scan times to more reasonable durations (11, 12).

### Ultrasound

Ultrasound is a medical imaging modality that uses high-frequency sound waves to generate images. This technique uses a piezoelectric transducer, which transmits sound waves by converting electrical energy into mechanical energy in the form of vibration. This transducer can also detect reflections of these transmitted sound waves which occur when the ultrasound enters media of different acoustic impedance. Acoustic impedance is a physical property of a material, defined as the resistance for the propagation of sound waves which varies as a function of the material density. These reflected waves—termed “echoes”—form the basis of image generation with ultrasound (13). When ultrasound waves travel through tissue with high acoustic impedance, a large amount of the incident acoustic energy is reflected and the tissues appear bright, or hyperechoic. On the other hand, ultrasound waves traveling through a tissue with low acoustic impedance results in greater transmission and less reflection of the incident energy, producing tissue that appears dark or hypoechoic.

Due to its low cost and relative accessibility compared to other medical imaging modalities, ultrasound has become a widely-adopted imaging tool used to screen for cancer, vascular disease, and trauma (14–16). It is also commonly used to aid with many image-guided procedures performed in real time. However, ultrasound is rarely used for longitudinal follow-up of diseases such as cancer, as the high degree of operator dependence and variability in image acquisition may result in underestimation of tumor size. This is due to the potential variations in imaging technique precluding consistent imaging for capture of maximal disease dimensions (17). For this reason, human clinical trials employ other cross-sectional imaging techniques such as CT and MRI, which are highly reproducible and less operator dependent than ultrasound.

### PET Imaging

**Figure 3:**
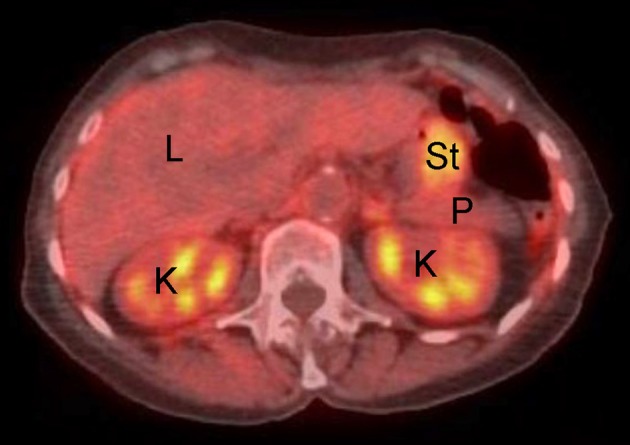
Example of normal fused abdominal PET-CT exam in human patient. L, liver; K, kidney; P, pancreas; St, stomach. Note background avidity of metabolically active liver and kidney (light orange color).

Popularized in 1990, PET is another imaging modality that has come into widespread use in clinical trials due to its ability to evaluate tumor metabolic response to therapy. Fluorine-18 (^18^F)-fluorodeoxyglucose (FDG) is a positron-emitting isotope which has become prevalent as a metabolic marker for imaging cancers of various origins (although this is not the only employed PET agent). As FDG is structurally similar to glucose, this radiotracer is taken up by cells much like the unlabeled sugar and undergoes the first step of glucose metabolism. After this step, however, the phosphorylated FDG molecule is trapped within a cell and, for all practical purposes, is not metabolized further. Given cancer cell preference for glycolysis as an energy source, this radiotracer and associated imaging technique allow for localization of neoplasms and metastatic disease that are highly glucose avid. While FDG is an effective tumor localizing agent, it should be noted that it is not specific for tumors alone, in that tissues with high background glucose uptake or excretion, such as the brain, kidneys, heart, and muscle, as well as inflamed tissues, may also exhibit high FDG signal. It should also be noted that not all tumors are FDG avid, and while some cancer types consistently exhibit moderate-to-high uptake, others are variable in their uptake, making the utility of this modality tumor-specific (18). The major advantage of PET imaging is the ability to image tissue viability rather than merely anatomy or structure; this provides useful functional oncologic information to guide clinical decision making. The main disadvantage of PET is its relatively low spatial resolution, a limitation which has been overcome in part through the co-registration of PET images with low dose CT images (termed “PET-CT”) to allow for better anatomic delineation of PET imaging observations (Figure 3). Recently, PET-MR imaging—which merges the tissue sensitivity and quantitative imaging features of MR imaging with the physiologic information of PET—has been investigated for its multimodal radiologic imaging capabilities (19).

## Rationale for Use of Medical Imaging in Clinical Trials

Contemporary medical imaging modalities are critical to the assessment of drug efficacy in oncology clinical trials. The non-invasive nature of radiologic imaging allows for serial monitoring of tumor stage throughout the treatment period, which, unlike more invasive tissue- or blood-based assays, avoids unnecessary patient trauma and allowing for use at more frequent intervals. Furthermore, clinically useful surrogate trial endpoints such as time-to-progression (TTP) and progression free survival (PFS) can be assessed by imaging, and have come into frequent use in drug trials as they may be observed shortly after initiation of therapy, and allow for an early assessment of treatment response; in contrast, use of overall survival (OS) as a primary endpoint requires protracted trial lengths to achieve, as well as relatively larger patient population required to properly power a study (20). These radiologic imaging outcome measures can thus help to reduce length and cost of trials, and also may allow trials to be adequately powered with smaller numbers of subjects, though these surrogate endpoints must be validated and demonstrated to be tightly correlated with clinical endpoints (21–23).

Additional benefits of medical imaging in clinical trials include image quantitation, automated processing and measurements, and real time transmission from trial sites to contracted research organizations that evaluate trial data. Quantitative measurement of medical imaging increases the accuracy of interpretation by eliminating subjective assessment of data, and the advent of automated and semi-automated image processing pipelines can reduce reader variability in trial analyses. The evaluation of large scale multicenter trial data requires rigorous standardization, in order to allow evaluation of patient data both longitudinally and across multiple sites. Variability can occur in both image acquisition and image interpretation, and so it is essential that standards are set a priori to minimize variation introduced by differences in scanner hardware, imaging parameters, contrast agent type and administration, or patient positioning. Thus, standards have been put in place, spanning regular calibration of scanners with phantom studies in order to account for performance drift, proper training of technologists to maintain consistency in patient positioning and acquisition, as well as proper blinding of readers in order to reduce bias (24).

## Diagnostic Schemes and Response Assessment Criteria: Examples From Human Clinical Care

In addition to rigorous standardization for image acquisition, standards must be set for interpretation of medical imaging data in order to enhance reporting consistency, reduce inter- and intra-reader variability, and increase comparability across investigations. For this reason, diagnostic schemes and response assessment criteria have been created to report findings using a systematic methodology and to provide universal descriptive verbiage such that reporting may be objective and reproducible regardless of reader.

### Diagnostic Schemes

**Figure 4:**
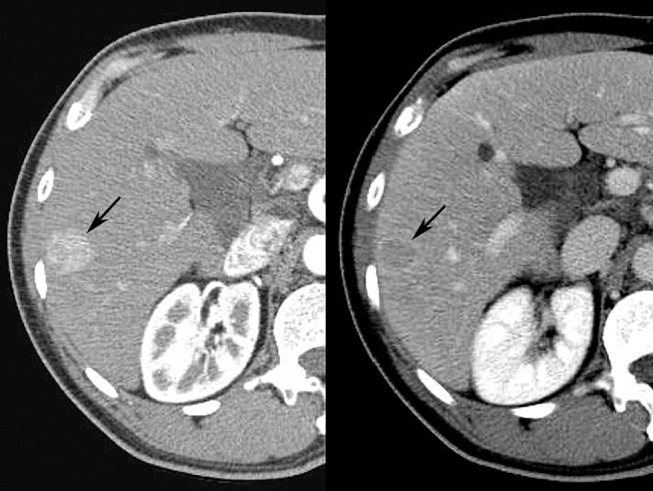
Arterial **(Left)** and venous **(Right)** phases of contrast enhanced CT scan performed in human patient demonstrate typical LI-RADS 5 mass (arrow), displaying typical arterial phase hyper enhancement, venous phase “washout,” and enhancing capsule.

Diagnostic classification systems allow for reliable and systematic interpretation of radiologic imaging studies (25). The American College of Radiology (ACR) supports several such schemes, including breast (BI-RADS), prostate (PI-RADS), and liver (LI-RADS) Imaging Reporting and Data System schemes, among others (19). Using liver imaging as an example, the ACR LI-RADS was developed in 2011 as a comprehensive classification system which standardizes radiological interpretation for liver cross-sectional imaging in patients at risk for primary liver cancer, or hepatocellular carcinoma (HCC) (26). This 5-point scale reporting system uses major and minor imaging features to classify a liver abnormality as definitely (LI-RADS 1) or probably (LI-RADS 2) benign, intermediate probability of malignancy (LI-RADS 3), probably malignant (LI-RADS 4), or definitely malignant (LI-RADS 5) (Figure 4). The classification of liver abnormalities is performed using features such as size, interval growth, arterial phase hyper enhancement, portal venous phase hypo enhancement, and capsular enhancement as major criteria, the presence of which favors the likelihood of malignancy. Utilization of LI-RADS enables the radiologist to employ specific descriptive terminology for consistent radiological reporting of liver abnormalities to meaningfully guide follow-up and/or treatment (27).

### Response Assessment Criteria

Response assessment schemes similarly allow for consistent and systematic interpretation of tumor response to treatment on radiologic imaging studies. A summary of various response assessment systems used in clinical oncology trials is presented in Table 2.

**Table 2:** Response outcome definitions for response assessment schemes.

**Measurement guidelines**	**WHO**	**RECIST 1.1**	**EASL**	**mRECIST**	**PERCIST**
Measurement guidelines	Change in sum of products of maximum bi-dimensional tumor diameters	Change in sum of maximum uni-dimensional tumor diameters; measurement of 5 target tumors with maximum of 2 tumors per organ	Change in sum of products of maximum bi-dimensional tumor diameters of viable (enhancing) tissue	Change in sum of maximum uni-dimensional tumor diameters of viable (enhancing) tissue; maximum of 2 liver tumors measured	Change in maximum SUL (SUV corrected for lean body mass); minimum target SUL should be >1.5x mean liver SUV + 2x SD liver SUV; target tumor with peak SUL at follow-up may be different from that selected at baseline
CR	Disappearance of all measurable tumors	Disappearance of all measurable tumors; short axis of all pathologic lymph nodes < 10 mm	Disappearance of all intra-tumoral enhancement in target tumors	Disappearance of all intra-tumoral enhancement in target tumors	Normalization of all target and non-target tumors to less than mean liver SUL, and equal or less than normal surrounding tissue
PR	≥50% reduction in sum of cross-products	≥30% reduction in sum of maximum diameters	≥50% reduction in sum of cross-products of viable (enhancing) tissue	≥30% reduction in sum of cross-products of viable (enhancing) tissue	≥30% reduction in peak SUL; absolute change must be ≥0.8 SUL
SD	Neither PR nor PD	Neither PR nor PD	Neither PR nor PD	Neither PR nor PD	Neither PR nor PD
PD	≥25% increase in sum of cross-products	≥20% increase in sum of cross-products	≥25% increase in sum of cross-products of viable (enhancing) tissue	≥20% increase in sum of cross-products of viable (enhancing) tissue	≥30% increase in peak SUL; absolute change must be ≥0.8 SUL

*WHO, World Health Organization; RECIST, Response Evaluation Criteria in Solid Tumors; EASL, European Association for the Study of the Liver; mRECIST, modified Response Evaluation Criteria in Solid Tumors; PERCIST, Positron Emission Tomography Response Criteria in Solid Tumors; CR, complete response; PR, partial response; PD, progressive disease; SD, stable disease; SUV, standardized uptake value; SUL, SUV lean*.

**Figure 5:**
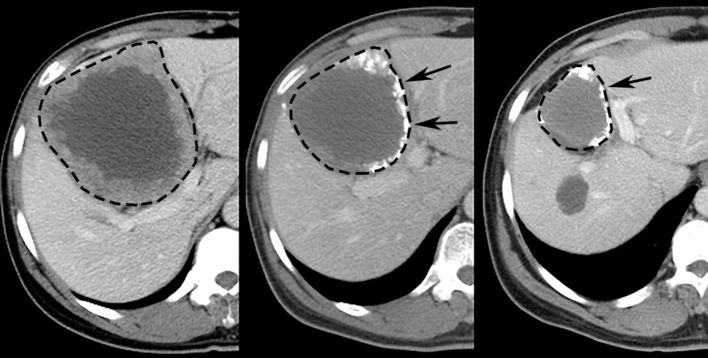
Representative images displaying RECIST response after TACE treatment of HCC in human patient. Pretreatment **(Left)** contrast-enhanced CT scan depicts 10.6 cm diameter right lobe liver tumor (dashed line). Sequential post-treatment contrast-enhanced CT scans **(Middle, Right)** reveal ensuing size reduction to 7.5 cm (29% reduction, RECIST SD) and 5.0 cm (53% reduction, RECIST PR) diameter, respectively; high attenuation material at tumor periphery (arrows) represents chemotherapy emulsion.

The original response criteria, first outlined by the World Health Organization (WHO) in the early 1980s, were anatomic in nature and based on the sum of the products of maximal perpendicular linear measurements of tumors. This guideline for response assessment has since been replaced by the Response Evaluation Criteria in Solid Tumors (RECIST)—created in 2000 and revised in 2009—which utilizes maximal unidimensional measurements and addresses some of the pitfalls and limitations of the original WHO guidelines. Although these response assessment criteria were generated during the era of cytotoxic chemotherapeutic agents, both remain in widespread use in clinical trials—with RECIST criteria in most widespread use in trials (28, 29)—and can be effective in situations where successful therapy results in a reduction in tumor size (30–33) (Figure 5).

**Figure 6:**
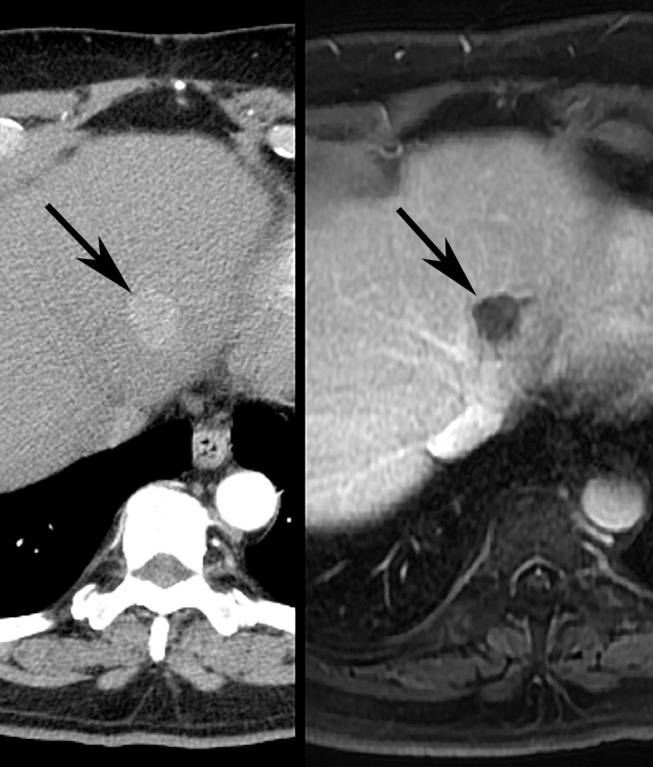
Illustrative images demonstrating EASL response after TACE treatment of HCC in human patient. Pretreatment **(Left)** contrast-enhanced CT scan depicts 2.0 cm diameter left lobe liver tumor (arrow). Post-treatment contrast-enhanced MR imaging study **(Right)** shows no residual enhancing component (arrow), consistent with EASL CR.

**Figure 7:**
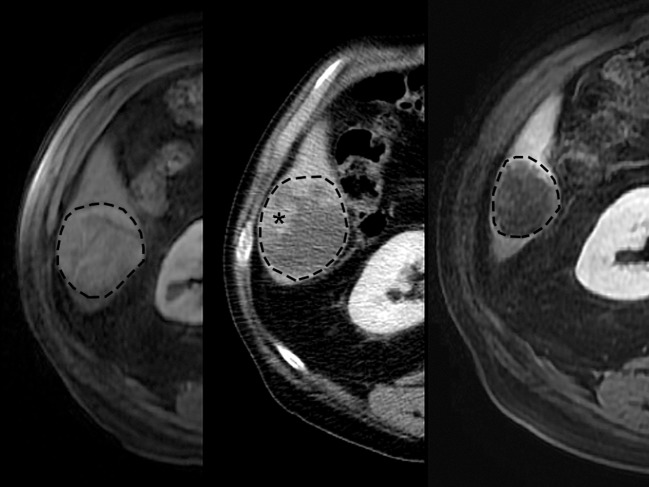
Typical images displaying mRECIST response after TACE treatment of HCC in human patient. Pretreatment **(Left)** contrast-enhanced MR imaging exam depicts 5.0 cm diameter right lobe liver tumor (dashed line). Contrast-enhanced CT scan **(Middle)** after first treatment demonstrates 1.5 cm residual enhancing tumor (asterisk) (70% reduction, mRECIST PR). Retreatment pursued, and contrast-enhanced MR imaging scan **(Right)** after second treatment demonstrates no residual enhancing tumor (100% reduction, mRECIST CR).

With the advent of new interventions such as immunotherapy and agents that selectively modulate specific molecular targets, it has become clear that tumor size changes are not the only or even the most effective indicator of treatment response for all cancer therapeutics. In patients with HCC, for example, locoregional therapies such as transarterial chemoembolization (TACE) induce tumor necrosis, often without change in tumor size (34–38). As such, a panel of HCC experts organized by the European Association for the Study of the Liver (EASL) generated a new set of response criteria which would take tumor necrosis into account. These EASL criteria would use reduction in viable tumor area, as determined by contrast enhancement on contrast-enhanced CT and MR imaging, as the primary method for evaluating treatment response in HCC (Figure 6). This was followed by a formal amendment to RECIST criteria in 2010—termed mRECIST—which would draw from the EASL definition of viable tumor, and simplify the measurement system from EASL criteria by using unidimensional linear summation (39, 40) (Figure 7). Several studies have since confirmed that both the EASL and mRECIST schemes may be better predictors of survival than WHO and RECIST criteria, respectively, for certain cancers (41–45), and may demonstrate better correlation with pathologic necrosis (46).

**Figure 8:**
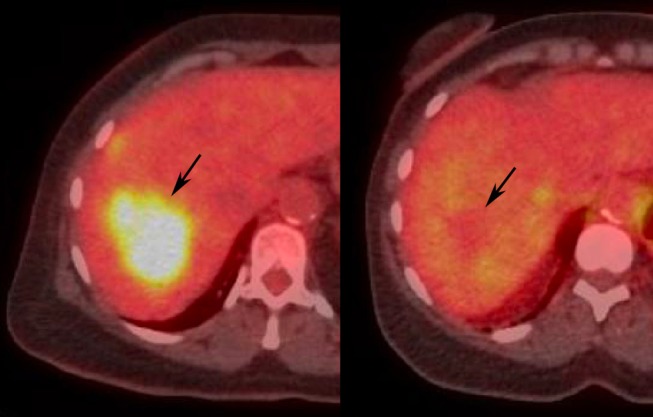
Illustrative images demonstrating PERCIST response after locoregional treatment of liver tumor in human patient. Pretreatment **(Left)** PET-CT demonstrates FDG avid liver tumor (arrow). Post-treatment **(Right)** PET-CT shows normalization of SUL (arrow), consistent with PERCIST CR.

FDG-PET radionuclide imaging has long been considered a potentially useful tool in detection of subclinical response to anti-tumor therapies. However, this technique also poses unique challenges in standardization of acquisition and reporting of results. The European Organization for Research and Treatment of Cancer (EORTC) proposed a common method for image acquisition, measurement of radiotracer uptake, and reporting of response data (47). The Positron Emission Tomography Response Criteria in Solid Tumors (PERCIST) scheme was later developed in 2009 and sought to further standardize the assessment of tumor metabolic response, and described detailed methods to allow longitudinal comparison of PET images (Figure 8). The PERCIST criteria utilizes a different method for image interpretation, and adds reporting instructions to clarify the time of imaging relative to initiation of therapy, as tumor radiotracer uptake can vary temporally depending on time from therapy (48, 49).

While EORTC and PERCIST have differences in implementation, the two criteria have demonstrated excellent agreement (50). Several studies have demonstrated that rapid reduction in FDG uptake in tumor, seen shortly after therapy, was correlated with later pathologic and radiographic response, with PET response far preceding any reduction in tumor size, while increases, no change, or modest reductions in FDG uptake after initiation of therapy were more likely to portend non-response (51–53). Importantly, studies have demonstrated that tumors exhibiting a partial metabolic response after initiation of therapy (as measured by PERCIST) were correlated with longer TTP and longer OS than those tumors that exhibited persistently high FDG uptake (54–57), and highlight the potential value of FDG-PET imaging and standardized metabolic response criteria such as PERCIST as a means for early identification of responders to therapy (58). Notably, combining anatomic imaging modalities (e.g., CT) with functional imaging data (e.g., PET) has shown value in assessing tumor response to therapy by leveraging both tumor size and metabolic changes toward optimal assessment of tumor response (59, 60).

## Animal Imaging for Preclinical Oncology Trials

### Challenges in Animal Imaging

All of the described radiologic modalities may be used for imaging in preclinical animal models of disease. However, animal radiologic imaging primarily differs from human clinical imaging in regards to the need for anesthesia; while human subjects are primarily imaged awake, animals are generally imaged under anesthesia to reduce gross motion during image acquisition. While examples exist of animals being trained to tolerate imaging procedures under intravenous sedation (61), as well as stereotactic techniques for restraining the body and head of smaller animals, longer imaging procedures such as MR imaging and PET acquisitions generally require anesthesia to prevent motion related image degradation. The use of anesthesia poses a unique set of challenges, and adds a layer of complexity for standardization in terms of animal handling, monitoring, and reporting. For instance, the reduced cardiac and respiratory drive caused by many anesthetics necessitates constant physiological monitoring (62). This becomes a logistical challenge inside an MR imaging suite, where neither radiofrequency emitting electronics (given risk for imaging artifacts) nor ferromagnetic materials (given risk for susceptibility artifacts, dislodgement, or near field heating) can be used. In addition to logistical challenges with monitoring during image acquisition, anesthetic agents can have variable effects on physiology, such as cardiac and respiratory depression, changes in cerebral blood flow and volume, and alterations in body temperature. While these physiological derangements may not affect structural imaging, they have been shown to affect radiotracer distribution and bioavailability, confounding the results of metabolic imaging modalities such as FDG-PET. In all, these considerations demonstrate the need for reporting of anesthetic agents used as well as animal handling protocols, as these factors can affect and confound imaging results (63–65).

## Existing Response Assessment Schemes for Animal Clinical Oncology Trials

With the growing use of large animal models of cancer, and the increasing number of prospective clinical trials using such models for assessing response to various therapies, there is an emerging need to standardize methodologies for evaluating tumor response in large animal models order to both improve the accuracy and consistency of reporting between various treatments and studies and increase the translatability to human clinical care. Currently, there is a paucity of published response evaluation criteria directly applicable in animal model systems. Given the lack of formal guidelines for assessment of response to therapy for solid tumors in animal models, the Veterinary Cooperative Oncology Group (VCOG) generated a consensus document based on recommendations from a subcommittee of the American College of Veterinary Internal Medicine (ACVIM) board certified veterinary oncologists in order to facilitate the design of a standardized protocol that would provide consistent, accurate, and reproducible reporting in therapeutic trials using animal oncology models. To that end, VCOG used the commonly implemented human response evaluation criteria RECIST as a framework for creating the canine response evaluation criteria in solid tumors (cRECIST v1.0), which is meant to provide specific guidelines for the measurement of solid tumors before, during, and after the initiation of therapy in prospective clinical trials using canine solid tumor models. This methodology is meant to be easily implemented, reproducible, and if widely adopted as anticipated, will standardize response assessment protocols to enable the comparison of current and future treatment strategies.

Recommendations from cRECIST follow many of the guidelines laid out from clinical RECIST. Some of these include baseline measurement of a tumor as close to the initiation of treatment, but no greater than two weeks prior to start of treatment. As with RECIST, the longest diameter in the plane of measurement should be recorded, and all subsequent measurements should be performed in the same plane of measurement. The minimum size of target tumors is 10 mm, and those masses falling below this threshold in the longest plane are considered non-measurable. If there exists more than one measurable target mass, a maximum of five target tumors should be reported with a maximum of two tumors per organ. Non-measurable and non-target tumors may be used in assessing overall tumor burden and should be reported as “present” or “absent” on follow-up, however for studies where tumor response is the endpoint, only subjects with measurable disease may be included. For studies where progression is the endpoint, the protocol must state whether subjects with non-measurable disease may be included. Much like RECIST, assessment of lymph nodes should report the longest diameter along the greater of either width or height at baseline (not length), and use a minimum size of 15 mm along this axis of measurement.

In terms of image acquisition, cRECIST recommends CT as the preferred imaging modality over MR imaging. This is due to the greater reproducibility in measurements; however, either may be used for measurement of tumors. Indeed, MR imaging provides superior soft tissue contrast, however the rapid image acquisition of CT paired with its high spatial resolution results in reduced motion during image acquisition and improved delineation of tumor boundaries, respectively, which are the likely reasons for the improved measurement reproducibility in CT. Ultrasound is generally not recommended due to the potential variability in acquisition, but given the cost of CT and MR imaging as well as the need for anesthesia (to reduce motion), cRECIST provides suggested guidelines for the use of ultrasound. These suggestions recommend that the same user perform assessments using the same machine at each time point in order to reduce inter-observer variation, a minimum target tumor diameter of 20 mm at baseline, and use of previously documented images to serve as a guide for subsequent imaging in order to allow for reassessment using previously used planes of image analysis (66).

While the canine cRECIST system represents a concrete example of standardization of imaging response assessment in animals, dogs represent clinical veterinary patients rather than biomedical animal model systems, and the translatability of the cRECIST scheme to biomedical animal models is unclear. At present, there are no available response evaluation criteria strategies for use in other large animal species, such as pigs. This fact is substantiated by the wide variation of response assessment methods used in published preclinical investigations (67), which span simple reporting of tumor diameter to description of percent tumor growth or involution, and which lack a common language for comparison across published studies. Development and validation of standardized tumor response assessment systems applicable in biomedical animal models represents an important barrier to broad employment of large animals in preclinical trials, and one which must be overcome if radiologic imaging is to be utilized for preclinical trial applications.

### Unmet Medical Imaging Needs for Large Animal Clinical Oncology Trials

While human clinical trials are the benchmark for advancing standard-of-care cancer therapeutics, the regulatory and financial burdens of clinical trials are—as previously noted—significant and time-consuming. Moreover, patient enrollment is challenging due to stringent eligibility criteria as well as competing clinical trials. Translational studies using validated animal models are thus critically essential in that they can efficiently and effectively undergo cohort clinical trial participation. This eliminates both accrual and logistical barriers to permit prospective early phase assessment of therapeutic modalities and to establish the validity of new technologies. Large animal models that faithfully recapitulate human patient tumor biology are particularly attractive for preclinical and co-clinical (parallel investigations in patients and animal cancer models to allow synchronization and real-time integration of preclinical and clinical efforts) trials for oncology. Given the integral role played by radiologic imaging in clinical trials, ensuring that medical imaging acquisition and interpretation is appropriately adapted to large animal cancer models is particularly important in developing the tools necessary for preclinical and co-clinical trial performance.

**Figure 9:**
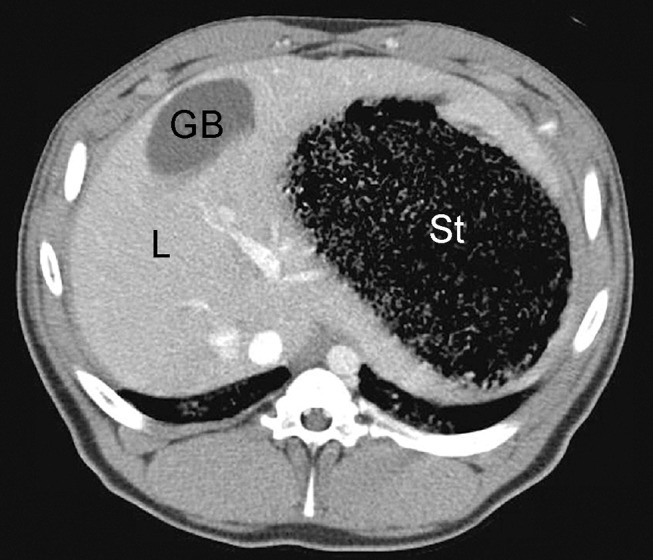
Example of normal contrast-enhanced porcine abdominal CT scan; L, liver; GB, gallbladder; St, stomach.

**Figure 10:**
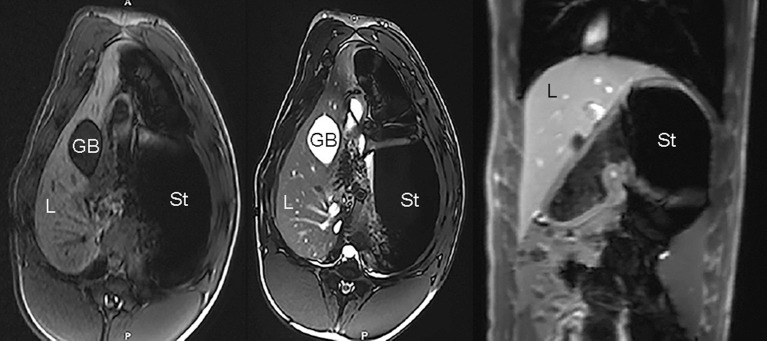
Example of unremarkable porcine MR imaging study: axial T1-weighted image **(Left)**, axial T2-weighted image **(Middle)**, and coronal T1-weighted post-contrast image **(Right)**; L, liver; GB, gallbladder; St, stomach.

First, large animal imaging protocols and workflows must be optimized to ensure rapid performance and efficient interpretation of imaging. To this end, recent efforts have supported the development of clinically translatable porcine liver CT and MR imaging protocols using human clinical imaging systems. This has resulted in a customized and tested clinical imaging workflow (68). The developed CT (Figure 9) and MR imaging (Figure 10) protocols demonstrate consistent and reproducible, high-resolution radiologic depiction of the liver which parallels human patient imaging. The protocols support the capability to use advanced radiological imaging for diagnostic surveillance and therapeutic outcomes analysis. Second, the development and widespread utilization of centralized cloud-based radiologic picture archiving systems aimed at facilitating large animal imaging data capture and sharing is necessary to parallel digital imaging capture and centralized interpretation used in human clinical trials. Third, with limited published experience reporting on large animal imaging and normal large animal radiologic anatomy, the range of normal findings must be defined through imaging of healthy subjects. Fourth, in the setting of disease, imaging findings for different large animal cancers must be validated against human cancer correlates, such that the specific imaging characteristics (location, morphology, vascularity, attenuation, signal, and avidity) of large animal disease parallel those seen in analogous clinical malignancies. Such validation may be pursued via systematic comparative radiology studies and radiologic-pathologic analyses. Fifth, the relative suitability of different imaging modalities for various large animal models, including dogs, primates, and pigs, requires exploration. Sixth, imaging benchmarks, diagnostic systems, and response assessment criteria need be extended to and standardized for all large animal platforms to allow investigators to make use of the range of available large animal models. To this end, current VCOG guidelines apply only to canine disease, though the use of pigs as a relevant large animal model is emerging (69). Such schemes must match validated systems which are recognized and employed by the clinical oncology community in order to enhance the relevance and applied translation to human clinical trials.

## Conclusions

The emergence of numerous large animal oncologic models has provided a means for the study of cancer pathophysiology, and has allowed drug developers to systematically evaluate the effectiveness of new therapies and treatment strategies while avoiding some of the regulatory and financial burdens associated with conducting human clinical drug trials. These models provide an alternative to small animal models, which often do not adequately mirror the complex physiology seen in human tumor biology. With the increasing potential for prospective clinical trials using large animal models, care must be taken to create and adhere to standardized protocols, in order to ensure reproducible results and to allow for the accurate comparison of study results across treatment strategies and sites. Clearly defined protocols for image acquisition and review are critical for the consistent handling of medical imaging data and objective assessment of response to therapy. Frameworks developed from human clinical trial image acquisition protocols, radiologic diagnostic schemes, and response assessment criteria can be tailored for use in large animal models, though care must be taken to ensure that such protocols are appropriately adapted to reflect nuances associated with specific models. Further validation of such animal models of disease and widespread adoption of universal protocols will help to streamline the drug development process and improve the care of human disease.

## Author Contributions

FF and RG: substantial contributions to the conception or design of the work, acquisition, analysis, or interpretation of data for the work, drafting the work or revising it critically for important intellectual content, and provide approval for publication of the content. RG: guarantor of integrity.

### Conflict of Interest Statement

The authors declare that the research was conducted in the absence of any commercial or financial relationships that could be construed as a potential conflict of interest.
